# Cognitive performance of healthy older adults and individuals with Alzheimer's Disease from a Latin American Country: the role of education on cognitive assessment batteries

**DOI:** 10.1002/alz70857_107243

**Published:** 2025-12-24

**Authors:** Leticia Fernanda Palma, Rebeca Carvalho Bom, Pedro Henrique Moreira Victoriano, Danilo Barroso de Sousa, Mateus Balleiro Bertoldo Garcia, Maria Clara Ribeiro Zucato, Karen Leticia Pulgatti, Vanessa Alexandre da Silva, Marina Mantellatto Grigoli, Sabrina Dorta de Oliveira, Cecilia Patricia Popolin, Patricia Regina Manzine, Marcia Regina Cominetti, Lucas Nogueira de Carvalho Pelegrini

**Affiliations:** ^1^ Federal University of São Carlos, São Carlos, SP, Brazil; ^2^ Federal University of Sao Carlos, São Carlos, Sao Carlos, Brazil; ^3^ Federal University of São Carlos, São Carlos, São Paulo, Brazil; ^4^ Federal University of Sao Carlos, São Carlos, Sao Paulo, Brazil; ^5^ Federal University of Sao Carlos, Sao Carlos, SP, Brazil; ^6^ Universidade Federal de São Carlos, São Carlos, SP, Brazil; ^7^ The Global Brain Health Institute, Trinity College Dublin, Dublin, Dublin, Ireland

## Abstract

**Background:**

Education is considered an important risk factor for neurocognitive disorders, especially among the Latin American population. It is also strongly associated with the cognitive performance of older adults and significantly correlated with cognitive reserve. This study aims to analyze the impact of education on cognitive performance of cognitively unimpaired older adults and people living with AD, and its relationship with the AD diagnosis in a Latin American sample living in Brazil.

**Method:**

This is a quantitative, cross‐sectional, descriptive, and correlational study involving 135 participants divided into two groups: cognitively unimpaired (*n* = 86) and individuals with Alzheimer's Disease (AD, *n* = 49). Assessments included a sociodemographic questionnaire, the Addenbrooke's Cognitive Examination‐Revised (ACE‐R), the Mini‐Mental State Examination (MMSE), and the word list from the CERAD battery. Data analysis was conducted using descriptive statistics, Student's t‐test, chi‐square test, and Pearson's correlation, with SPSS v.29.0.0 and a significance level of *p* ≤0.05.

**Result:**

Most participants were female (65.7%), married (60.4%), retired (72.9%), and physically active (53.7%). The group comparison analysis revealed the AD group was older (79.6 ± 7.6 years; t=‐8.59; *p* <0.001), had lower educational levels (6.7 ± 4.7 years; t=5.93; *p* <0.001), and performed worse on ACE‐R (42.3 ± 19.1; t=15.04; *p* <0.001), MMSE (16.5 ± 5.7; t=13.64; *p* <0.001), and the CERAD word list (immediate recall: 7.6 ± 4.4; t=12.23; *p* <0.001; delayed recall: 0.7 ± 1.6; t=6.08; *p* <0.001; recognition: 6.3 ± 3.1; t=5.69; *p* = 0.002). Considering the variability in education, groups were then stratified into low education (≤8 years) and high education (>8 years). The lower education group (≤8 years) had more individuals with AD (X^2^ = 24.95; *p* <0.001), lower family income (X^2^ = 27.95; *p* <0.001), and practice less physical activity (X^2^ = 14.68; *p* <0.001). Correlation analysis indicated a negative relationship between cognitive assessment performance and both age and educational level.

**Conclusion:**

Our findings highlight the influence of education on cognitive performance and its association with cognitive reserve in older adults. Lower education levels were linked to poorer cognitive outcomes and a higher prevalence of AD, emphasizing the need for lifelong educational opportunities to mitigate cognitive decline, especially in the Latin American context. Future studies should further explore targeted interventions for individuals with limited education.

